# A ribozyme that uses lanthanides as cofactor

**DOI:** 10.1093/nar/gkad513

**Published:** 2023-06-16

**Authors:** Kevin J Sweeney, Xu Han, Ulrich F Müller

**Affiliations:** Department of Chemistry and Biochemistry, University of California San Diego, La Jolla, CA, USA; Department of Chemistry and Biochemistry, University of California San Diego, La Jolla, CA, USA; Department of Chemistry and Biochemistry, University of California San Diego, La Jolla, CA, USA

## Abstract

To explore how an early, RNA-based life form could have functioned, *in vitro* selection experiments have been used to develop catalytic RNAs (ribozymes) with relevant functions. We previously identified ribozymes that use the prebiotically plausible energy source cyclic trimetaphosphate (cTmp) to convert their 5′-hydroxyl group to a 5′-triphosphate. While these ribozymes were developed in the presence of Mg^2+^, we tested here whether lanthanides could also serve as catalytic cofactors because lanthanides are ideal catalytic cations for this reaction. After an *in vitro* selection in the presence of Yb^3+^, several active sequences were isolated, and the most active RNA was analyzed in more detail. This ribozyme required lanthanides for activity, with highest activity at a 10:1 molar ratio of cTmp : Yb^3+^. Only the four heaviest lanthanides gave detectable signals, indicating a high sensitivity of ribozyme catalysis to the lanthanide ion radius. Potassium and Magnesium did not facilitate catalysis alone but they increased the lanthanide-mediated k_OBS_ by at least 100-fold, with both K^+^ and Mg^2+^ modulating the ribozyme's secondary structure. Together, these findings show that RNA is able to use the unique properties of lanthanides as catalytic cofactor. The results are discussed in the context of early life forms.

## Graphical Abstract

**Graphical Abstract:**
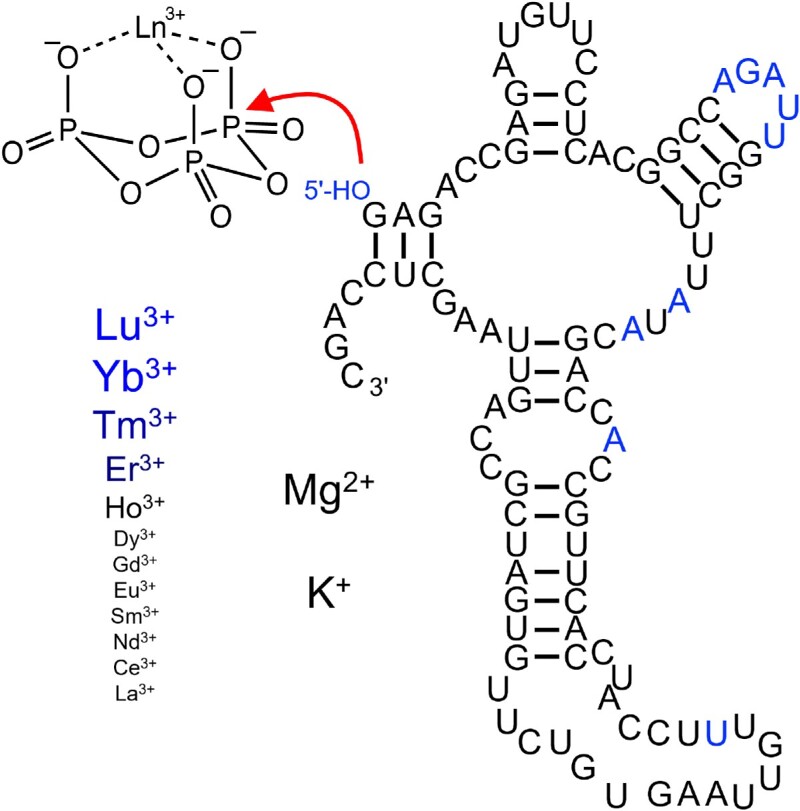

## INTRODUCTION

The RNA world hypothesis describes an early life form in which RNA served as the genome and as the only genome-encoded catalyst (1–4). To study how an RNA based organism could function, researchers have developed catalytic RNAs (ribozymes) in the lab, using *in vitro* selection (5–7). The most central activity for self-replication is ribozyme-catalyzed RNA polymerization (8), which requires chemically activated nucleotides. Nucleoside 5′-triphosphates (NTPs) could have been used as chemically activated nucleotides because NTPs are used in all of biology as activated nucleotides, with their involvement in diverse, fundamental metabolic and signaling processes pointing to an ancient origin (9,10) and because known chemical pathways could have led to prebiotic activation of nucleotides in the form of 5′-triphosphates (11–13).

Any self-replicating molecular system requires a source of energy from the environment to drive structure formation, dictated by the second ‘law’ of thermodynamics (14). In other words, for any molecular system, the free energy released by the chemical conversion of that energy source (or the free energy provided by physical forces such as light, or temperature changes) needs to exceed the free energy that is required to replicate the structured, information-rich system. Cyclic trimetaphosphate (cTmp) could have served as such an early, external energy source because it is is prebiotically plausible (15,16), and it can serve as polyphosphorylation reagent to generate NTPs (11–13,17), which are then able to drive polymerization or other reactions due to the highly exergonic hydrolysis of pyrophosphate bonds. This energy source can also be used by ribozymes, as shown by an *in vitro* selection of ribozymes that react their 5′-hydroxyl group with cTmp and generate 5′-triphosphates (18). Such 5′-triphosphorylated ribozymes were then able to energetically drive otherwise endergonic ligation between 5′-phosophate and 3′-hydroxyl groups. This previous selection was performed with Mg^2+^ cofactors because Mg^2+^ is known to aid ribozyme catalysis for a wide range of activities (7,19–23), and because Mg^2+^ likely existed at concentrations around 10–20 mM in the prebiotic ocean (24,25).

Our interest to explore lanthanides as ribozyme cofactors stems from their ability to activate the phosphorus atoms of cTmp for nucleophilic attack by water (26). Importantly, the rate of this catalysis is enhanced if the lanthanide is coordinated not only by the negatively charged oxygens of cTmp but also with additional ligands such as the carboxy groups of EDTA (ethylenediaminetetraacetate) or NTA (nitrilotriacetate). We hypothesized that analogously, the lanthanide should also accelerate the nucleophilic attack of RNA 5′-hydroxyl groups to the cTmp phosphorus atoms, and that a beneficial effect similar to that of EDTA and NTA could also be mediated by ribozymes, which often coordinate a Mg^2+^ ion at their catalytic site to support acid/base catalysis (27–31). Lanthanide (III) ions are excellent catalysts for many reactions in water also due to their near-neutral hydrolysis constants of coordinated water molecules, and their high water exchange rate (32). Together, these ideas motivated us to explore the potential of lanthanides as ribozyme cofactors.

It is possible that lanthanide cofactors could have been used in an RNA world because they can be sufficiently abundant and accessible in specific environments, and because they are used as enzyme cofactors in contemporary organisms. First, lanthanides are not as rare as the name ‘rare earth elements’ suggests: Their abundance in the Earth's crust is in the range of several mass ppm, similar to cobalt and molybdenum (33). Due to their unique chemical behavior, they are highly enriched in specific ores and pegmatites (34). While lanthanide minerals usually have low mobility, their mobility can increase via chelation by organic compounds in the environment (35). Second, in today's biology, several methylotrophic bacteria use lanthanides as cofactors for methanol dehydrogenases that are closely related to enzymes using Ca^2+^ as cofactor (36–38). When both lanthanides and calcium are available then the lanthanide using enzyme is used preferentially, likely because the lanthanide provides greater catalytic potential due to its stronger Lewis acidity and higher ligand turnover (39). The gene encoding the lanthanide dependent methanol dehydrogenase is widespread in several coastal marine environments (40). Therefore, the lanthanide's unique catalytic properties provide enough evolutionary benefit in some environments to make them useful as catalytic cofactors in today's biology, and perhaps even in early stages of life.

To test whether ribozymes could use lanthanides as cofactor, we performed an *in vitro* selection for self-triphosphorylation ribozymes as described previously (18), but in the absence of Mg^2+^ and in the presence of the lanthanide Yb^3+^. After five rounds of selection, many isolated clones showed detectable activity with Yb^3+^. The ribozyme with the highest activity was characterized in more detail. This ribozyme used Yb^3+^ for catalysis and gained >100-fold in activity from the additional presence of K^+^ and Mg^2+^, which modulated the ribozyme's secondary structure. Catalysis was mediated only by the heaviest lanthanides with the smallest ion radii. Together, these results showed that ribozymes are able employ the strong Lewis acidity of lanthanides for use as catalytic cofactors.

## MATERIALS AND METHODS

### *In vitro* selection

The *in vitro* selection was performed essentially as described (18), except that the triphosphorylation reaction step was performed with 3 mM Yb^3+^ and 10 mM cTmp instead of 100 mM Mg^2+^ and 50 mM cTmp. The Yb^3+^ concentration of 3 mM was chosen because this concentration did not result in detectable RNA degradation at neutral pH and 22°C over several hours, while some degradation was visible under the same conditions at 10 mM Yb^3+^. The starting DNA library was synthesized based on a custom ultramer single-stranded DNA (Integrated DNA Technologies, IDT) with the sequence 5′-**GCTGGAGCTTAACTGGCG**-(N150)-**AACATCTCGGTCTC**GACTG-3′ (lower strand) with hand-mixed preparation of phosphoramidites for the randomized region to reduce nucleotide bias. This sequence was used as template for PCR amplification with PCR primers 5′-*AATTTAATACGACTCACTATA*GGGCGGTCTCCTGACGAGCTAAGCGAAACTGCGGAAACGCAGTC**GAGACCGAGATGTT**-3′ and 5′-**GCTGGAGCTTAACT**-3′ to generate the double-stranded DNA library (italicized T7 promoter, underlined hammerhead ribozyme, bold constant regions). The hammerhead ribozyme cleaved itself off co-transcriptionally, generating a 5′-hydroxyl group at the RNA library. After PAGE purification, 100 nM library RNA was incubated with 50 mM Tris/HCl pH 8.3, 3 mM Yb(Tf)_3_, 10 mM Na_3_cTmp, 150 mM NaCl and 5 mM NaOH (to account for the pH drop due to cTmp chelating metal ions) in a volume of about 100 mL at room temperature. The RNA was then ethanol precipitated, desalted by size exclusion chromatography (P30 spin-columns, Bio-Rad) and ethanol precipitated again. The recovered RNA was ligated to a biotinylated oligonucleotide (5′-biotin-d(GAACTGAAGTGTATG)rU-3′) using the R3C ligase ribozyme (41) with its arms adjusted to anneal to the 5′-constant region of the RNA library and to the biotinylated oligonucleotide (800 nM library RNA, 1000 nM ligase ribozyme, and 1200 nM biotinylated oligonucleotide in 100 mM KCl, 100 mM Tris/HCl pH 8.5 and 2 pM of randomized library RNA transcribed without a hammerhead ribozyme, which means that it had a 5′-triphosphate). The triphosphorylated RNA was included for the first 4 selection rounds to reduce the number of PCR cycles in each selection round such that no PCR artifacts (i.e. shorter amplicons) were detected. This 5′-triphosphorylated RNA contained the same N150 randomized region as the starting library. Therefore, it generated a low amount of ‘noise’ in the selected sequences but it did not introduce a bias from a specific sequence that could generate a false positive. The ligation products were captured on streptavidin-coated magnetic beads (Promega Z5481) and washed with water, 50 mM NaOH, and again water. After elution with 96% formamide at 65°C for 5 min the RNA was ethanol precipitated, reverse transcribed with the 3′ PCR primer (Superscript III Reverse Transcriptase, Invitrogen), and PCR amplified in two steps. The first PCR used 5′-primer 5′-GAACTGAAGTGTATGTGAGACCGAGA-3′ and 3′-primer 5′-GCTGGAGCTTAACT-3′, and the number of required PCR cycles was used to monitor the progress of the selection. The second PCR used the same PCR primers as the generation of the initial DNA library to complete one round of selection. The selection to optimize the N20 sequence in the truncated ribozyme 15 (Figure 2) was performed identically, only the conditions during the selection step were adjusted to aid activity of ribozyme clone 15. Specifically, the pH was lowered to pH 7.3, and the concentrations of Yb^3+^ and cTmp were adjusted to 6 mM each (round 1), 3 mM each (round 2), 1 mM each (round 3) and 0.3 mM each (round 4).

### Self-triphosphorylation activity assays

The assay for self-triphosphorylation activity of individual ribozymes was performed essentially as described (18). The DNA sequence containing the T7 promoter, hammerhead ribozyme, and the sequence of a specific, selected ribozyme sequence was transcribed by runoff transcription and the RNA was purified by denaturing PAGE. The standard triphosphorylation conditions before optimizing conditions for ribozyme 51 included 50 mM Tris/HCl pH 7.3, 150 mM NaCl, 3 mM Yb(Tf)_3_,10 mM Na_3_cTmp and 5 μM ribozyme RNA. After incubation at 22°C for 3 h, an aliquot of 2 μl was removed and added to 8μl of a pre-mixed solution such that the 10 μl combined solution contained 100 mM Tris/HCl pH 8.0, 100 mM KCl, 5.6 mM Na_2_EDTA, 1 μM of R3C ligase ribozyme, 1 μM of the ribozyme RNA, 1 μM of 5′-[^32^P] radiolabelled oligonucleotide 5′-d(GAACTGAAGTGTATG)rU-3′. The mixture was heated 2 minutes to 65°C, then cooled at ∼0.1°C/s to 30°C to anneal the ligase ribozyme with its substrates. To start the ligation reaction, an equal volume of a solution containing 50 mM MgCl_2_, 4 mM spermidine and 40% (w/v) PEG 8000 was added, incubated for 3 h at 30°C, and ethanol precipitated. The products were separated on 7 M urea 10% PAGE, exposed to phosphorimaging screens, detected by scanning on a Typhoon phosphorimager (GE), and quantified using the rectangle tool in the Quantity One software (Bio-Rad). Because triphosphorylation ribozyme, ligase ribozyme, and biotinylated oligonucleotides were equimolar the fraction of the ligated, short radiolabeled oligonucleotide was equal to the fraction of ligated ribozyme RNA, and therefore a measure of the fraction of self-triphosphorylated ribozyme. In the first screen of 27 selected RNA clones under selection conditions (at pH 8.3), only few clones showed activity. However, the activity of the most active ribozyme was much higher at pH 7.3, where about half of the clones showed activity (Supplementary Figure S2). Therefore the conditions for the initial triphosphorylation assays were pH 7.3 (including 6 mM Yb(Tf)_3_, 6 mM Na_3_cTmp, 50 mM HEPES/NaOH pH 7.3, and 150 mM NaCl, for 3 h at 22°C) before the concentrations of Yb^3+^, cTmp, Na^+^/K^+^ and Mg^2+^ as well as temperature and pH were optimized.

### SHAPE probing

The ribozyme secondary structure was studied by SHAPE chemical probing with 1-methyl 7-nitro isatoic acid anhydride (1M7), which reacts with 2′-hydroxyl groups of flexible nucleotides (42). Final concentrations during the probing were 50 mM MOPS/KOH pH 6.8, 330 μM Yb(Tf)_3_ (if included) and 3.3 mM cTmp (if included), 200 nM ribozyme 51, and the specified amounts of KCl and MgCl_2_. These compounds were dissolved in 49 μl water and immediately mixed with 1μl of a solution containing either 100 mM 1M7 in dry DMSO or dry DMSO without 1M7. After incubation at 25°C for three minutes the RNA was ethanol precipitated, reverse transcribed using the manufacturer's instructions (Superscript III, Invitrogen) using an 8 nucleotide 5′-[^32^P] radiolabeled DNA primer complementary to the ribozyme's 3′-terminus. This was the shortest primer that resulted in full extension. Reverse transcription products were heated with 750 mM NaOH at 80°C for 5 minutes to hydrolyze RNA, buffered to pH 5 by the addition of two equivalents of acetic acid over NaOH, and ethanol precipitated. Reaction products were separated by 7 M urea 10% polyacrylamide gelelectrophoresis, exposed to phosphorimager screens, scanned on a Typhoon Phosphorimager (GE), and quantified using the rectangle tool in the software Quantity One (Bio-Rad). For each band, the signal in the lane without 1M7 was subtracted from the signal in the lane with 1M7. Reverse transcription stops at the nucleotide before the 1M7 modified nucleotide, which allowed assigning the bands to positions in the ribozyme, and thereby generating a SHAPE reaction profile for each experiment. Since each experiment used slightly different amounts of radioactivity, each profile was normalized, and the averages and standard deviations from three experiments were reported. The secondary structures were based on computational predictions by unafold (43); during these predictions, nucleotides were constrained as unpaired if they showed high SHAPE signals.

## RESULTS

**Figure 1.**
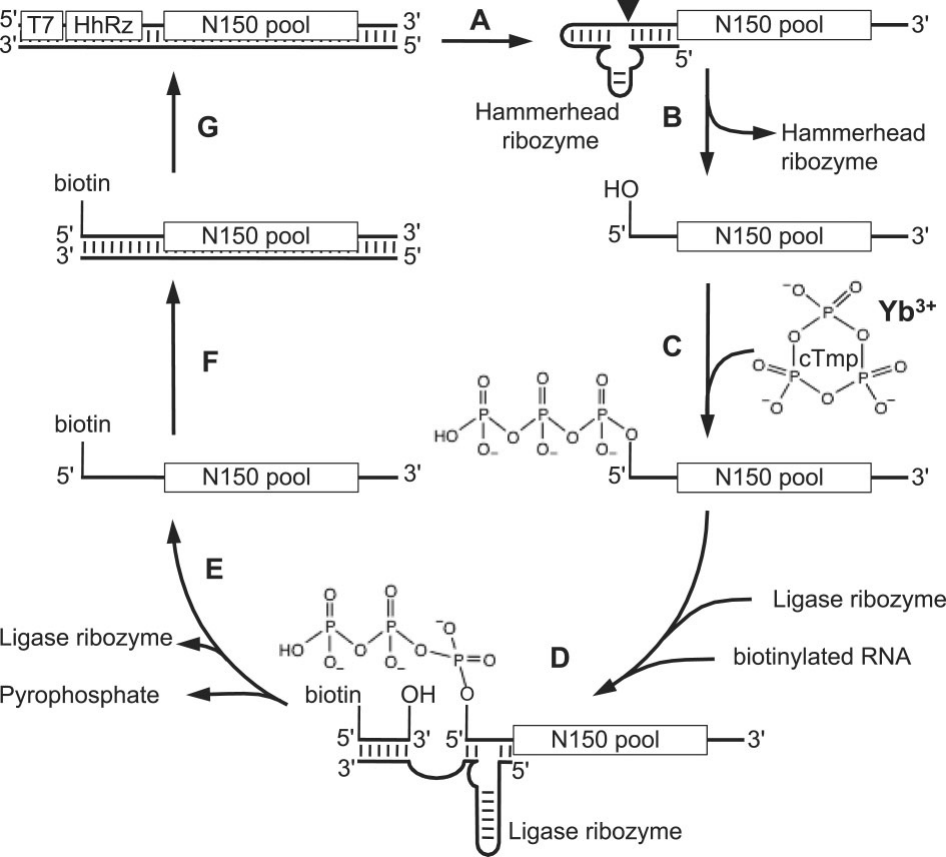
Scheme for the *in vitro* selection, modified after (18). The double-stranded library DNA contained a T7 promoter, a hammerhead ribozyme, and 150 randomized positions flanked by constant regions. (**A**) Transcription by T7 polymerase led to (**B**) co-transcriptional self-cleavage of the hammerhead ribozyme and generated the RNA library with 5′ hydroxyl groups. (**C**) Incubation of these RNAs with cTmp, Yb^3+^, and 50 mM Tris/HCl pH 8 allowed some RNAs to catalyze the nucleophilic attack of their 5′-hydroxyl group onto cTmp, and thereby self-triphosphorylate. (**D**) Incubation with the R3C ligase ribozyme (41) covalently linked 5′-triphosphorylated RNAs to biotinylated oligonucleotides. (**E**) Biotinylated sequences were isolated with streptavidin-coated magnetic beads. The selected sequences were then (**F**) reverse transcribed and (**G**) PCR amplified to regenerate the double-stranded DNA library, now enriched for sequences catalyzing self-triphosphorylation.

To test whether ribozymes would be able to use lanthanides as cofactors we first tried to identify lanthanide-using ribozymes from a library that was previously selected from random sequence by 8 rounds of selection in the presence of 100 mM Mg^2+^ and 50 mM cTmp (18). This library shows activity also at 51 mM Mg^2+^ and 1 mM cTmp (44) and contained hundreds of different ribozyme clusters (45). To identify lanthanide-using ribozymes from this library, the library was subjected to four rounds of selection in the absence of Mg^2+^, but in the presence of 3 mM Yb^3+^ and 5 mM cTmp (Figure 1). Four selection rounds were previously sufficient to enrich active ribozymes from a library with more than 10^14^ different sequences to dominate the population (18,45), therefore we expected that lanthanide-using ribozymes would dominate the library within one or at most two selection rounds from the pre-selected library. However, even after five selection rounds in the presence of Yb^3+^ no ribozyme activity was detected with Yb^3+^ (Supplementary Figure S1), suggesting that the previously selected library with hundreds of Mg^2+^-using ribozymes did not contain a single ribozyme that was able to alternatively use Yb^3+^ as cofactor.

To test more generally whether Yb^3+^ could be used by ribozymes as cofactor for triphosphorylation we conducted a new *in vitro* selection, starting from random sequence. The starting library was the same, ‘round 0′ library with 150 randomized positions that was used as starting library for the previous selection of ribozymes in the presence of Mg^2+^ ions. The selection in the presence of Yb^3+^ ions used this library with an effective complexity of 2.0 × 10^14^ different sequences. After four rounds of selection, the library appeared to be dominated by active sequences because the required number of PCR cycles after reverse transcription decreased sharply (Supplementary Figure S2A). The selection was continued until selection round 8 with shorter incubation time (initially 3 h reduced to 2 min) to select for the most active ribozymes.

To identify active ribozymes, 29 clones were arbitrarily chosen from selection round 8 and analyzed for self-triphosphorylation activity. For this assay, individual sequences were generated with a 5′-hydroxyl group, incubated with cTmp and Yb^3+^, then ligated to an equimolar concentration of a 5′-[^32^P] radiolabeled oligonucleotide using the R3C ligase ribozyme (41). Because only 5′-triphosphorylated RNAs could be ligated, the gel-shifted fraction of radiolabeled oligonucleotide informed about the extent of self-triphosphorylation (Supplementary Figure S2B). The fraction of ligated oligonucleotide was equal to the fraction of ligated ribozyme because these two RNAs were employed in equimolar concentrations. When the incubation with cTmp was conducted under selection conditions (pH 8.3), only weak activity was detected. However, activity was significantly higher at pH 7.3, especially when 150 mM NaCl was added (final 6 mM YbCl_3_, 6 mM Na_3_cTmp, 50 mM HEPES/KOH pH 7.3, 150 mM NaCl). Here, at least 19 of the 29 clones showed activity, and one clone showed more than 10-fold higher activity than any of the others. This clone 15 was chosen for further analysis.

**Figure 2.**
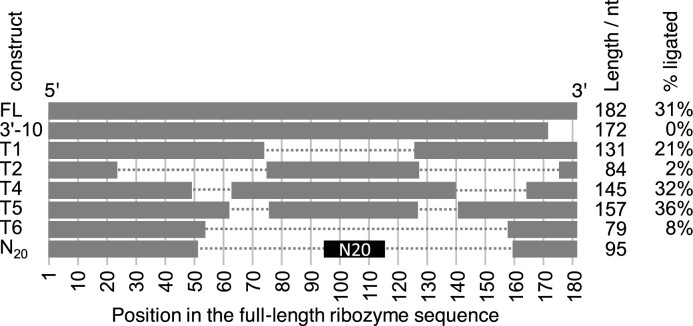
Truncation analysis of the most active ribozyme clone 15. For each ribozyme construct, the name (left column), the graphical schematic of the truncation (grey bars, to scale) and the remaining length is shown, as well as the activity of each variant (right column). Only the first 3′-truncation variant is shown (3′-10) because no 3′-truncation variant showed activity. Breaks in the horizontal bars for constructs T1–T6 denote internal deletions. The replacement of sequence 53–159 with an N_20_ randomized sequence is annotated in black. No activity is listed for the N_20_ construct because this construct describes the library with many different sequences and activities.

To identify the minimal size of ribozyme clone 15 (182 nucleotides in length), its 3′-terminus was truncated in 10-nucleotide increments. However, even the truncation of 10 nucleotides abolished activity (Figure 2), suggesting that the 3′-terminus was required for activity. This finding matched the computationally predicted secondary structure of clone 15 (43), which positioned the ribozyme 3′-terminus close to the reactive site at the 5′-terminus. The same structure prediction was used as guide to delete internal regions of the ribozyme. Indeed, removing three different segments of the region between position 49 and 163 (T1, T4 T5 in Figure 2) retained most of the activity but activity was reduced significantly when all three regions were removed simultaneously (T6 in Figure 2). To determine whether this central region could be replaced by a shorter fragment, we inserted 20 randomized nucleotides between position 53 and 159 of the ribozyme, and subjected this library to an additional four rounds of selection, with each successive selection round decreasing the concentration of Yb^3+^ and cTmp. Each round required only 7–8 PCR cycles to amplify the selected sequences, suggesting that the majority of sequences in the initial N_20_ library mediated activity. Twenty-three clones were arbitrarily chosen from the fourth round of this selection and analyzed for activity (Supplementary Figure S3). The most active variant, ‘ribozyme 51′ with a length of 95 nucleotides, was chosen for further analysis. While ribozyme 15 and ribozyme 51 are related in sequence, ribozyme 51 is much smaller and was used for the further experiments.

**Figure 3.**
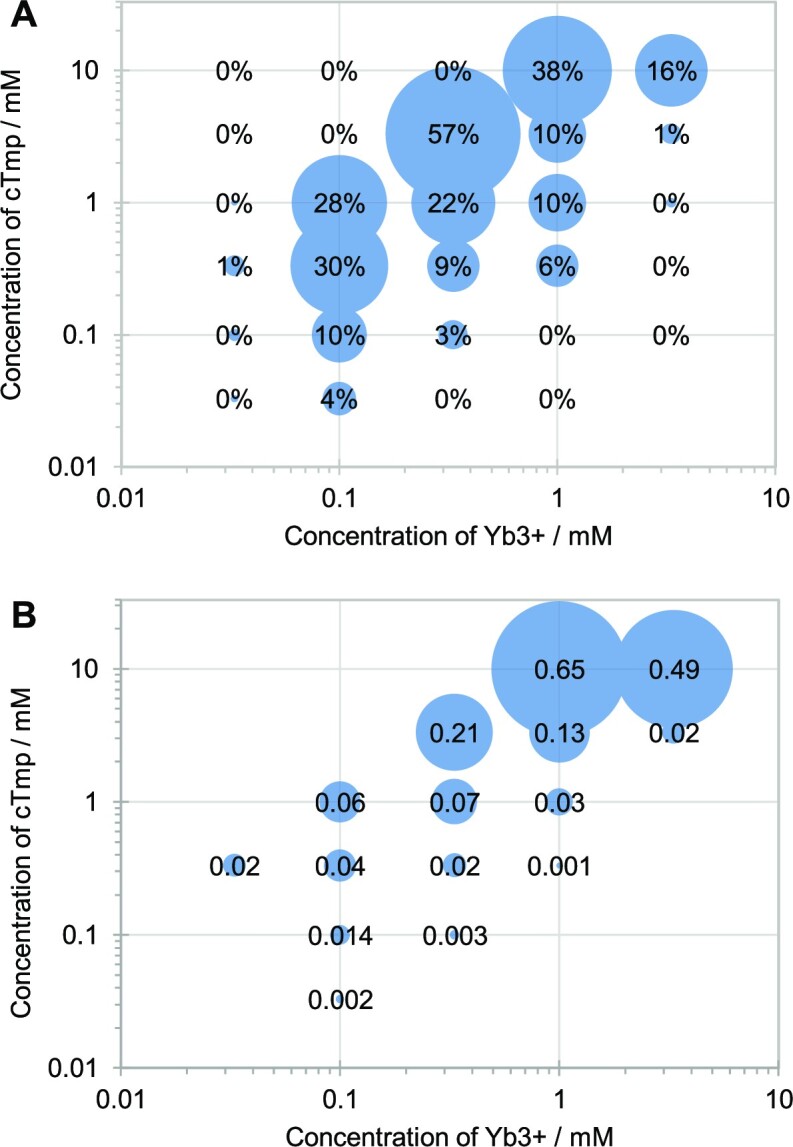
Ytterbium and trimetaphosphate concentration dependence of ribozyme 51 activity with 50 mM HEPES/KOH pH 7.3 and 150 mM NaCl, at 22°C. (**A**) The signal (percent ligated) of the reaction after 9 h reaction time (average from three experiments). (**B**) The rate of the reaction (min^−1^), based on single-exponential curve fits to the data (Supplementary Figure S4). Rates are only shown for reactions with an amplitude of at least 1% to avoid background noise. Averages from three experiments were used as basis for the curve fits.

To identify the optimum reaction conditions for ribozyme 51, the concentrations of Yb^3+^ and cTmp were co-varied, and reaction kinetics recorded (Figure 3). The highest reaction amplitude (57%) was reached at 3 mM cTmp and 0.3 mM Yb^3+^, while the fastest rate (0.65 min^−1^) was seen at 10 mM cTmp and 1mM Yb^3+^ (full data shown in Supplementary Figure S4). Yb^3+^ concentrations as low as 33 μM led to detectable activity. The ratio of cTmp:Yb^3+^ was optimal at 10:1 for most tested combinations, resulting in the highest amplitude and the fastest rate. The drop in activity at higher ratios was most dramatic at Yb^3+^ concentrations of 0.1 and 0.3 mM, where a cTmp:Yb^3+^ ratio of 10:1 led to yields of 28% and 57%, while a cTmp:Yb^3+^ ratio of 30:1 led to yields of 0.0% and 0.1%. This behavior is consistent with the ribozyme binding Yb^3+^ as a complex with two or three cTmp molecules. More cTmp molecules may prevent the ribozyme from coordinating Yb^3+^, and the coordination of only one cTmp molecule (with three negative charges) by one Yb^3+^ ion would lead to an uncharged, insoluble complex (26).

**Figure 4.**
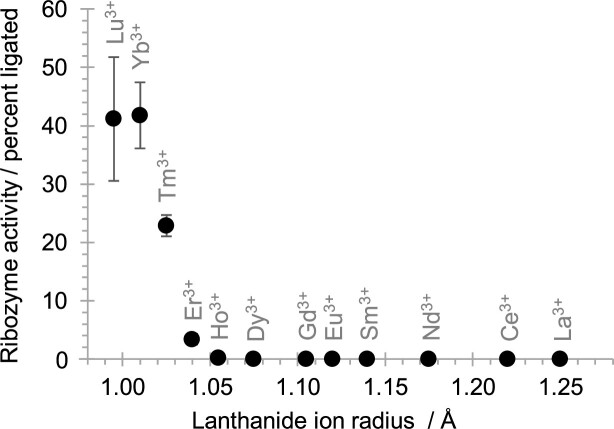
Ribozyme 51 activity with different lanthanide (III) cations. Ribozyme 51 activity is shown as a function of the radius of the lanthanide (III) ions, with each tested lanthanide labeled. Reactions were conducted at 0.1 mM lanthanide^3+^, 1 mM cTmp, 50 mM HEPES/KOH pH 7.3, 150 mM NaCl and 2 h reaction time. Error bars represent standard deviations from triplicate experiments, and are smaller than the symbols if not visible.

To test whether other lanthanide (III) ions could replace Yb^3+^ as cofactor we tested twelve different lanthanides at a concentration of 0.1 mM Ln^3+^ and 1 mM cTmp (Figure 4). Ribozyme activity was maximal with Lu^3+^ and Yb^3+^ (ion radii of 0.995 and 1.010 Å, respectively), half-maximal for Tm^3+^ (1.025 Å), weak with Er^3+^ (1.040 Å), and barely detectable with Ho^3+^ (1.055 Å). Lighter lanthanides (ion radii larger than 1.055 Å) did not lead to detectable catalysis, thereby revealing a strong sensitivity of ribozyme catalysis to the lanthanide (III) ion radius.

**Figure 5.**
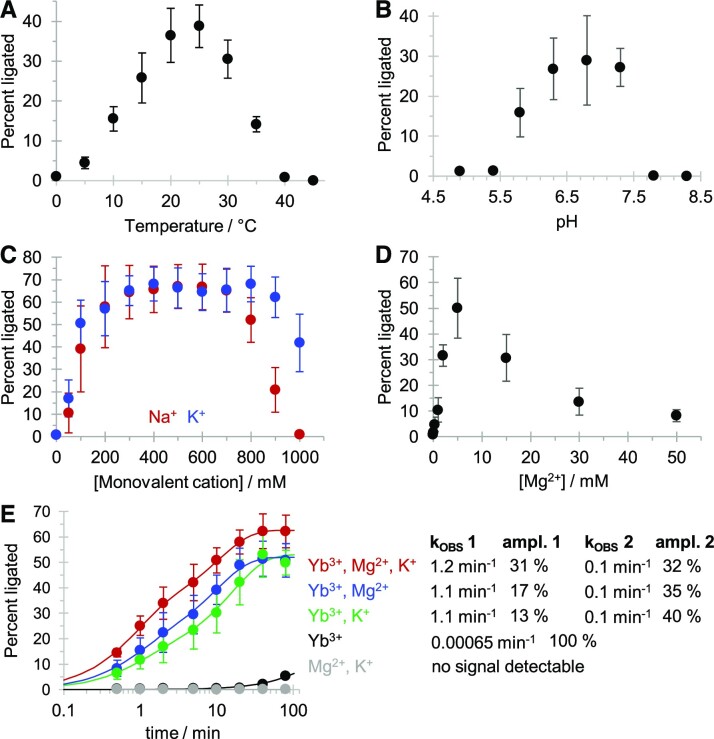
Optimization of reaction temperature, pH, Na^+^/K^+^ and Mg^2+^ for ribozyme 51. (**A**) Effect of reaction temperature between 0°C and 50°C. Reactions were executed at 0.1 mM Yb^3+^, 1 mM cTmp, 50 mM HEPES/KOH pH 7.3, 150 mM NaCl and 20 minutes reaction time. (**B**) Effect of reaction pH, at pH 4.9 and 5.3 (NaOAc/HOAc), 5.8 and 6.3 (MES/NaOH), 6.8 (MOPS/NaOH), 7.3 and 7.8 (HEPES/NaOH), and 8.3 (Tris/HCl). Reactions were executed at 0.1 mM Yb^3+^, 1 mM cTmp, 50 mM buffer, 150 mM NaCl, 25°C, and 20 min reaction time. (**C**) Effect of varying concentrations of KCl (blue) or NaCl (red) added to the triphosphorylation reaction. Reactions were executed at 0.1 mM Yb^3+^, 1 mM cTmp, 50 mM HEPES/KOH pH 7.3, 25°C and 20 minutes reaction time. (**D**) Effects of varying concentrations of MgCl_2_ added to the triphosphorylation reaction. Reactions were executed at 0.1 mM Yb^3+^, 1 mM cTmp, 50 mM HEPES/KOH pH 7.3, 25°C and 20 minutes reaction time. (**E**) Reaction kinetics with different combinations of 500 mM KCl, 5 mM MgCl_2_, and 0.1 mM Yb^3+^, all at 1 mM cTmp, 50 mM HEPES/KOH pH 7.3, and 25°C. Colors represent reactions with Yb^3+^, K^+^, Mg^2+^ (red), Yb^3+^ and Mg^2+^ (blue), Yb^3+^ and K^+^(green), Yb^3+^ (black), and Mg^2+^ and K^+^ (grey). Curved lines represent double-exponential equations fitted to the data by least squares fitting, with the rates and amplitudes for each condition given to the right. Note that the reaction time in the graph is displayed in logarithmic order. Error bars in all panels represent standard deviations from triplicate experiments.

The variation of reaction temperature, reaction pH, and concentrations of other cations identified an optimum for each parameter (Figure 5). The temperature showed an optimum around 25°C, similar to the temperature at which the *in vitro* selection of the ribozyme was performed (22°C). The pH was optimal in the neutral range between 6.3 and 7.3, which is close to the hydrolysis constant of the inner sphere lanthanide-coordinated water/hydroxyl around 7.7 (32). Under these conditions (50 mM MOPS / NaOH pH 6.8, 3.3 mM cTmp, 0.33 mM Yb^3+^, 25°C) the reaction yield was highest with 300–700 mM NaCl or KCl, or 5 mM MgCl_2_, with a rise in reaction yield from about 5% to 68%, and 50%, respectively. The beneficial effect of Yb^3+^, Mg^2+^ and K^+^ was confirmed in the reaction kinetics, where different combinations of 500 mM KCl, 5 mM MgCl_2_, and 0.33 mM Yb^3+^ were employed (Figure 5E). The ribozyme was inactive without Yb^3+^, indicating that the other cations were not able to replace Yb^3+^ in catalysis. Ribozyme activity was maximal with all three cations (Yb^3+^, K^+^, Mg^2+^) and decreased in the order of Yb^3+^/K^+^/Mg^2+^ > Yb^3+^/Mg^2+^ ∼ Yb^3+^/K^+^ > Yb^3+^. Curve fitting of the kinetics in the presence of Mg^2+^ and/or K^+^ required two exponentials, which resulted for all three reactions in a fast rate around 1.1 min^−1^ and a slow rate around 0.1 min^−1^. The highest amplitude for the fast rate was in the presence of all three ions. These results suggested that the ribozyme requires Yb^3+^ for catalysis and is dependent on K^+^ (or Na^+^) and Mg^2+^ to fold into its active structure. While the functional roles of these cations may be overlapping (for example, in shielding the negative charges) the data show that each ion has a sufficiently distinct role to be required for optimal activity.

**Figure 6.**
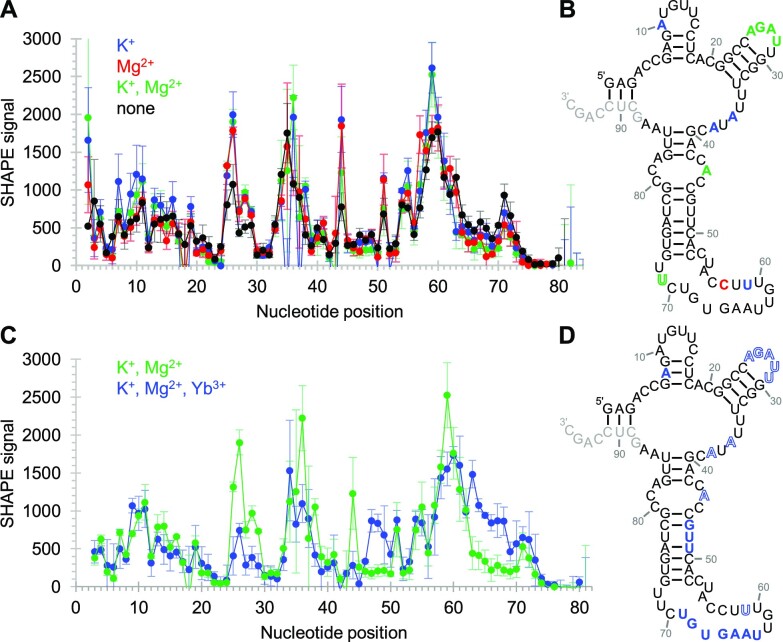
Secondary structure analysis of ribozyme 51 using SHAPE chemical probing. Error bars represent standard deviations from triplicate experiments. (**A**) Dependence of SHAPE reactivity on the presence of K^+^ and Mg^2+^. Colored symbols indicate the presence of K^+^ (blue), Mg^2+^ (red), and K^+^ and Mg^2+^ (green) during the probing reaction. (**B**) Secondary structure prediction, constrained by SHAPE probing data. The positions where the SHAPE accessibility was changed outside of error by the addition of cations were colored for K^+^ (blue), Mg^2+^ (red), and both K^+^ and Mg^2+^ (green). (**C**) Dependence of SHAPE reactivity on the presence of both Yb^3+^ and cTmp in the presence of K^+^ and Mg^2+^ (blue), compared to K^+^ and Mg^2+^ alone (green). (**D**) Secondary structure as in (A), showing the influence of the Yb^3+^/cTmp complex. Nucleotides colored in solid color had increased SHAPE reactivity, whereas letters colored in outlines had decreased SHAPE reactivity due to the Yb^3+^ / cTmp complex. Nucleotides in light grey were used for annealing the reverse transcription primer of the SHAPE assay.

The ribozyme's secondary structure, and its dependence on Yb^3+^, cTmp, K^+^ and Mg^2+^ was studied by SHAPE probing in the absence and presence of these ions (Figure 6). The shown structure was consistent with the SHAPE probing data except positions 53–72, which may form a more complicated arrangement (see below). The influence of 500 mM K^+^ and 5 mM Mg^2+^ on the secondary structure (Figure 6A) was studied without Yb^3+^ or cTmp by SHAPE probing at 50 mM MOPS/NaOH pH 6.8. Therefore, the influence of K^+^ and Mg^2+^ shows the status before Yb^3+^/cTmp substrate binding. The data showed that positions 25–28 were *more* accessible in the presence of K^+^ or Mg^2+^, which is consistent with K^+^ and Mg^2+^ modulating the structure to present the loop 25–28 for coordination with Yb^3+^/cTmp complex (see below). While K^+^ increases the flexibility of the four positions 10, 36, 38, and 59, Mg^2+^ makes nucleotide 57 more flexible and nucleotide 71 less flexible. These observations suggest specific binding sites that mediate these ion's support in the ribozyme's structure and catalysis (Figure 5E).

The addition of Yb^3+^ and cTmp to the ribozyme in presence of K^+^ and Mg^2+^ led to a rigidification of five nucleotide positions 25–29, position 36 near the central loop (Figure 6C,D); this may represent the formation of the catalytic site at the Yb^3+^/cTmp complex. Positions flanking the region 57–60 (i.e. 47–49 and 63–69) became more flexible upon the addition of the Yb^3+^/cTmp complex. Since this region co-incides mostly with the 20-nucleotide insert (positions 53–72), and since the re-selection of this insert identified many active sequences (Supplementary Figures S2 and S3), the flexible positions 47–49 and 63–69 may represent linker regions that allow the protected nucleotides 52, 53 and 56 to remain in place while the ribozyme undergoes a conformational change due to binding of the Yb^3+^/cTmp complex. In contrast, cTmp alone did not influence the SHAPE accessibility in the absence of Yb^3+^, which suggested that the ribozyme binds cTmp only as a complex with Yb^3+^, consistent with the observed co-dependence of ribozyme activity on cTmp and Yb^3+^ (Figure 3).

**Figure 7.**
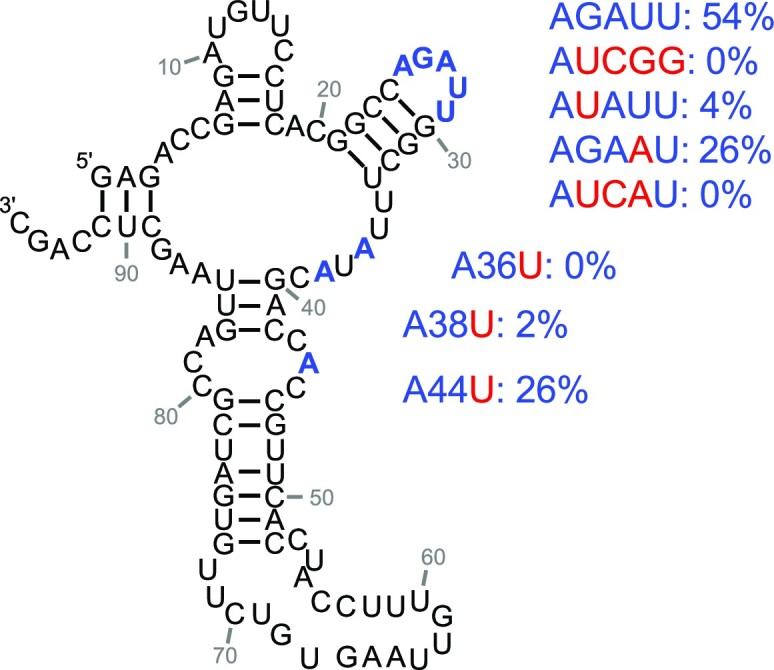
Effects of mutations at ribozyme 51 positions that were affected by Yb^3+^/cTmp binding (Figure 6C, D). The loop with positions 25–29, and the three bulged A’s are highlighted in blue. The mutations are highlighted in red, with the corresponding ribozyme activity given to the right. Values are averages from three experiments. Further details are shown in Supplementary Figure S5.

Since the ribozyme's loop at positions 25–29 was affected by Yb^3+^/cTmp binding (figure 6C, D), we tested whether this loop is indeed important for the ribozyme's function (Figure 7). To do this, we introduced mutations into the loop and measured the ribozyme's activity. The loop sequence AGAUU was converted to Aucgg (mutated positions lowercase), AuAUU, AGAaU or AucaU. All four variants showed a decrease in ribozyme activity, with AGAaU maintaining about half of its activity, AuAUU generating about a 10-fold decrease in activity, and the activity mediated by Aucgg and AucaU below the detection limit. In addition, we tested the importance of three bulged adenosines (A36U, A38U, A44U) that also changed SHAPE accessibility upon Yb^3+^/cTmp binding (Figure 6C, D). While the mutation of the bulged A44 retained half of the ribozyme's activity, mutations A36U and A38U showed a >10-fold reduction in activity. These data confirm that positions 36, 38, and the loop at positions 25–29 are central to the ribozyme's function, and may be directly involved in Yb^3+^/cTmp binding.

To test whether ribozyme 51 could function on a substrate *in trans*, the loop at positions 10–15 was split in half, and the stem extended to a length of 8 base pairs so that the ribozyme could use it as binding site for a 14-nucleotide long substrate RNA (Supplementary Figure S6). However, this did not result in detectable activity even after 16 hours under optimal conditions. In contrast, in side-by-side experiments the trans-reacting variant of ribozyme TPR1e (44) gave near-complete substrate conversion after 1 minute reaction time under its own optimal conditions. These results suggested that the lanthanide-using ribozyme 51 required the stem-loop from nucleotides 7–18 for its activity and cannot be easily converted to the trans-format.

**Figure 8.**
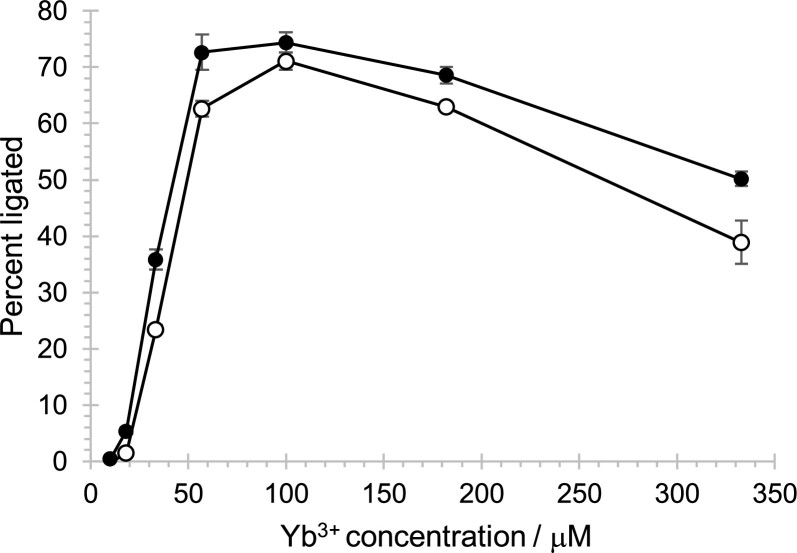
Influence of the inserted 20 nucleotide region at different Yb^3+^/cTmp concentrations. Two constructs were compared: Ribozyme 51 with a selected N20 region (black filled circles), and ribozyme T6, which is lacking this region (empty circles). The constructs are shown in Figure 2. The ribozyme activity (percent ligated) is shown as a function of the concentration of Yb^3+^, which was added with a 10-fold stoichiometric excess of cTmp. Error bars are standard deviations from triplicate experiments.

To better understand the importance of the inserted region 53–72 (20 nucleotides inserted into the T6 construct with an internal truncation, Figure 2), the activity of ribozyme 51 with these 20 nucleotides, and the T6 variant without these 20 nucleotides were measured under optimized conditions (50 mM MOPS/NaOH pH 6.8, 500 mM KCl, and 5 mM MgCl_2_, and a 1:10 molar ratio of Yb^3+^:cTmp) across a range of Yb^3+^/cTmp concentrations (Figure 8). The 20 nucleotide insert increased ribozyme activity across the tested Yb^3+^/cTmp concentrations, with a similar increase at 33 μM and 330 μM Yb^3+^. The observation that the increase was similar at sub-saturating, and saturating concentrations of Yb^3+^/cTmp suggested that the 20-nucleotide insert did not increase the ribozyme's affinity for the Yb^3+^/cTmp complex but that it generally stabilized the ribozyme's active conformation. Additionally, these results showed that the ribozyme can show detectable activity with as little as 18 μM Yb^3+^ and 180 μM cTmp. The sigmoid shape of the curve suggested cooperative behavior of added cTmp and Yb^3+^, consistent with the idea that cTmp and Yb^3+^ are bound as a complex.

## DISCUSSION

In this study, we describe the *in vitro* selection, and analysis of a ribozyme that uses Yb^3+^ as a catalytic cofactor. Three pieces of evidence support the idea that Yb^3+^ participates in catalysis: First, even without the context of a ribozyme, lanthanides strongly coordinate the three negatively charged oxygens of cTmp and activate the phosphorus atoms for nucleophilic attack (26). Second, Yb^3+^ is required for ribozyme-mediated self-triphosphorylation catalysis, even in the presence of Mg^2+^, Na^+^ or K^+^ (Figure 5E). Third, ribozyme activity is co-dependent on the concentration of cTmp and Yb^3+^ with an optimal 10:1 ratio of [cTmp] : [Yb^3+^] (Figure 3). This is expected if cTmp and Yb^3+^ are bound as a complex at the catalytic site.

The lanthanide-using ribozyme 51 showed a pH optimum around pH 6.8 (Figure 5B), not far from the hydrolysis constant of the inner sphere lanthanide-coordinated water/hydroxyl around 7.7 (32). This is consistent with a lanthanide-coordinated water/hydroxide being used in Lewis acid/base catalysis of the reaction. In contrast, previously characterized self-triphosphorylation ribozymes that use Mg^2+^ as cofactor show an increase of *k*_OBS_ beyond pH 8, suggesting that for these ribozymes the rate-limiting step is the deprotonation of a group with a much higher p*K*_A_, such as the ribozyme's 5′-hydroxyl group (18). This different use of the catalytic cations could explain the failure to select lanthanide-using ribozymes from a library that was previously selected in the presence of Mg^2+^ ions (Supplementary Figure S1). In contrast, a previously generated RNA-cleaving DNAzyme that was selected in the presence of Mg^2+^ was active with lanthanides as cofactors (46). These data suggest that the promiscuity in the use of Mg^2+^ versus lanthanides differs between the catalyzed reaction and / or the catalyst (triphosphorylation vs. RNA cleavage). The sensitivity of ribozyme 51 to the lanthanide ion radius is consistent with the size-selective coordination seen for lanthanides in minerals (47), protein enzymes (48) and deoxyribozymes (49).

Contemporary organisms use lanthanides as cofactor for several methanol dehydrogenases (MDHs), which are evolutionarily related to calcium-using MDHs (36,38). While the calcium-using MDHs oxidize methanol to formaldehyde, the lanthanide-using MDHs (with a preference for Ce^3+^) are catalytically more efficient and oxidize methanol to the less toxic formic acid (37). In both enzymes, the cations are coordinated at the catalytic site using the keto oxygens O5 and O7 as well as the aromatic N6 of pyrroloquinoline quinone (PQQ). However, these enzymes differ in their additional coordination of the cations by amino acid side chains as well as the backbone geometry near the catalytic site (37). When both Ca^2+^ and Ce^3+^ are accessible, methanotrophic organisms seem to preferentially use the lanthanide-dependent MDH (39). To scavenge lanthanides in environments of low lanthanide abundance, the protein lanmodulin binds lanthanides with picomolar affinity (50). The evolution of lanthanide using MDHs and lanmodulin underscore the high value of lanthanide cations as catalytic cofactors in biological enzymes.

Could lanthanides have been used as catalytic cofactors in prebiotic RNA catalysis? In contrast to Mg^2+^, which was likely present at about 10 mM concentration in the Hadean ocean (25), lanthanide^3+^ ions would have been present in much lower concentrations, and the lanthanides are often hard to mobilize. However, the lanthanides in modern soils (around 1–100 mg/kg) can be mobilized by lanthanide-coordinating humic acids (33,35), and the lanthanide's poor bioavailability under neutral pH is enhanced by acidic environments such as the volcanic mud pot water where biological lanthanide use was first discovered, and which contained lanthanides at 2–3 micromolar concentration (51). Since volcanic environments were likely among the few land forms on early Earth (52) there could have been ecological niches in which early RNA-based life forms used ribozymes with lanthanides as catalytic centers. However, the current study is focused on the broader picture to explore the chemical space accessible for RNAs to achieve efficient catalysis. In addition to the eight metal ions in the main groups (Li, Na, K, Mg, Ca, Sr, Ba, Pb) and seven transition metal ions (Mn, Fe, Co, Ni, Cu, Zn, Cd) that have been known to serve as cofactors for ribozymes (53–56), this study demonstrates with four additional elements (Lu, Yb, Tm, Er) that even the strong Lewis acidic lanthanides can be used as cofactors for ribozymes.

## Supplementary Material

gkad513_Supplemental_File

## Data Availability

All data necessary for the reproduction of the described study are described in the manuscript text, figures, and supplemental figures.
