# Supplementary material for: A ribozyme that uses lanthanides as cofactor
Source: Nucleic Acids Res. 2023 Jun 16;51(14):7163–73. doi: 10.1093/nar/gkad513 (PMC10415125; doi:10.1093/nar/gkad513)
Supplement: gkad513_Supplemental_File [file gkad513_supplemental_file.pdf]

Supplementary Figures

A Ribozyme that uses Lanthanides as Cofactor

Kevin J. Sweeney, Xu Han, and Ulrich F. Müller

Figure S1:
Failure to isolate Yb^{3+} using self-triphosphorylation ribozymes from a library containing >300 ribozymes active with Mg^{2+}.

Figure S2:
Successful enrichment of Yb^{3+} using self-triphosphorylation ribozymes from a high-complexity library containing 150 randomized positions.

Figure S3:
Sequences and biochemical analysis of 23 ribozyme variants with different N20 inserts.

Figure S4:
Full data for the activity dependence of ribozyme 51 con the concentrations of cTmp and Yb^{3+}.

Figure S5:
Details on the analysis of individual mutations in ribozyme 51, including mutations in the loop positions 25-29 and three bulged adenosines at positions 36, 38, and 44.

Figure S6:
Test of ribozyme 51 to act in trans on a short RNA oligonucleotide.

Figure S1

A

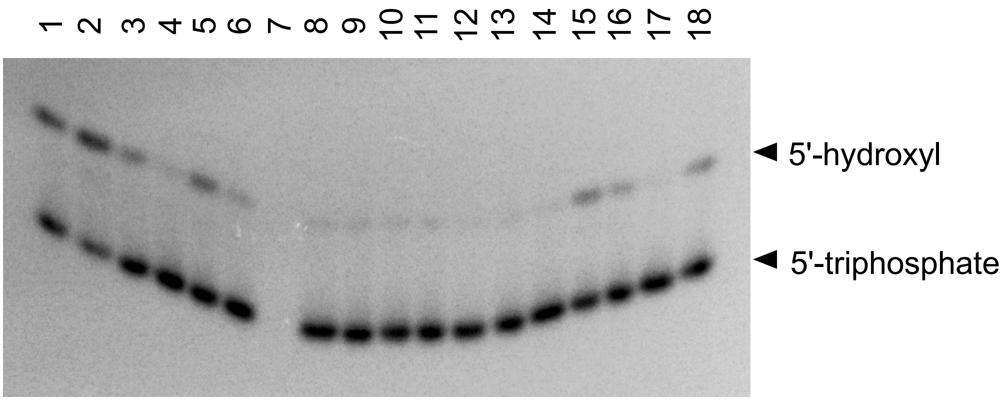

B

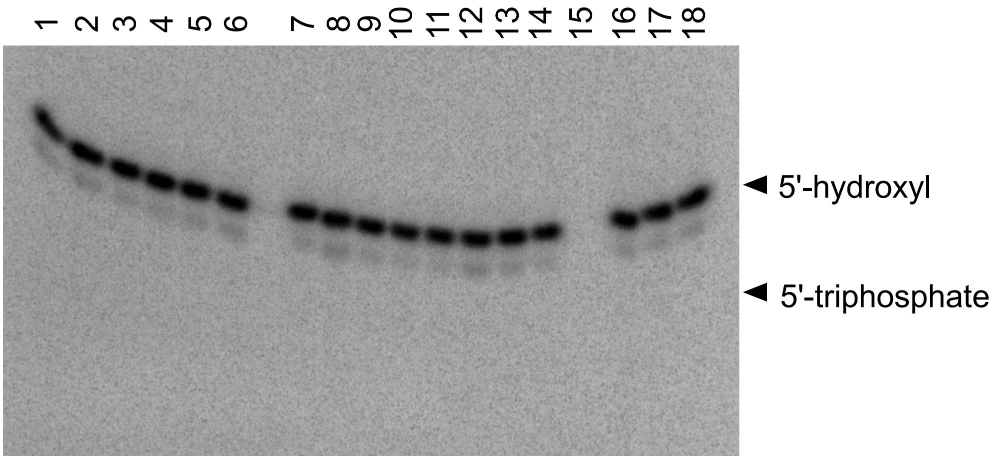

Figure S1: Activity of ribozyme clones (1-18) arbitrarily chosen from a library that was first selected for 8 selection rounds in the presence of Mg^{2+} (Moretti & Müller 2014), then for 5 selection rounds in the presence of Yb^{3+}. Shown are phosphorimages of radiolabeled RNAs separated by 20% PAGE. The assay was performed as described (Moretti & Müller, 2014). In short, ribozymes were radiolabeled internally by transcription with α-[^{32}P]ATP, reacted with cTmp, then processed with a catalytic DNA (DNAzyme). This DNAzyme cleaved the first 8 nucleotides of the ribozymes, generating an 8-nucleotide fragment that carried either a 5'-hydroxyl group or a 5'-triphosphate. Due to its additional 3.5 negative charges, the 5'-triphosphated RNA octanucleotide migrates faster than the 5'-hydroxyl octanucleotide. **(A)** Activity in the presence of Mg^{2+}. The sample for ribozyme 7 was lost during sample processing. **(B)** Activity of the same ribozyme clones in the presence of Yb^{3+}. Several lanes were not used due to malformed sample loading pockets. The sample for ribozyme 15 was lost during sample processing. The band migrating only slightly faster than the 5'-hydroxyl RNA in (B) is an n-1 product and not due to 5'-triphosphorylation.

Figure S2

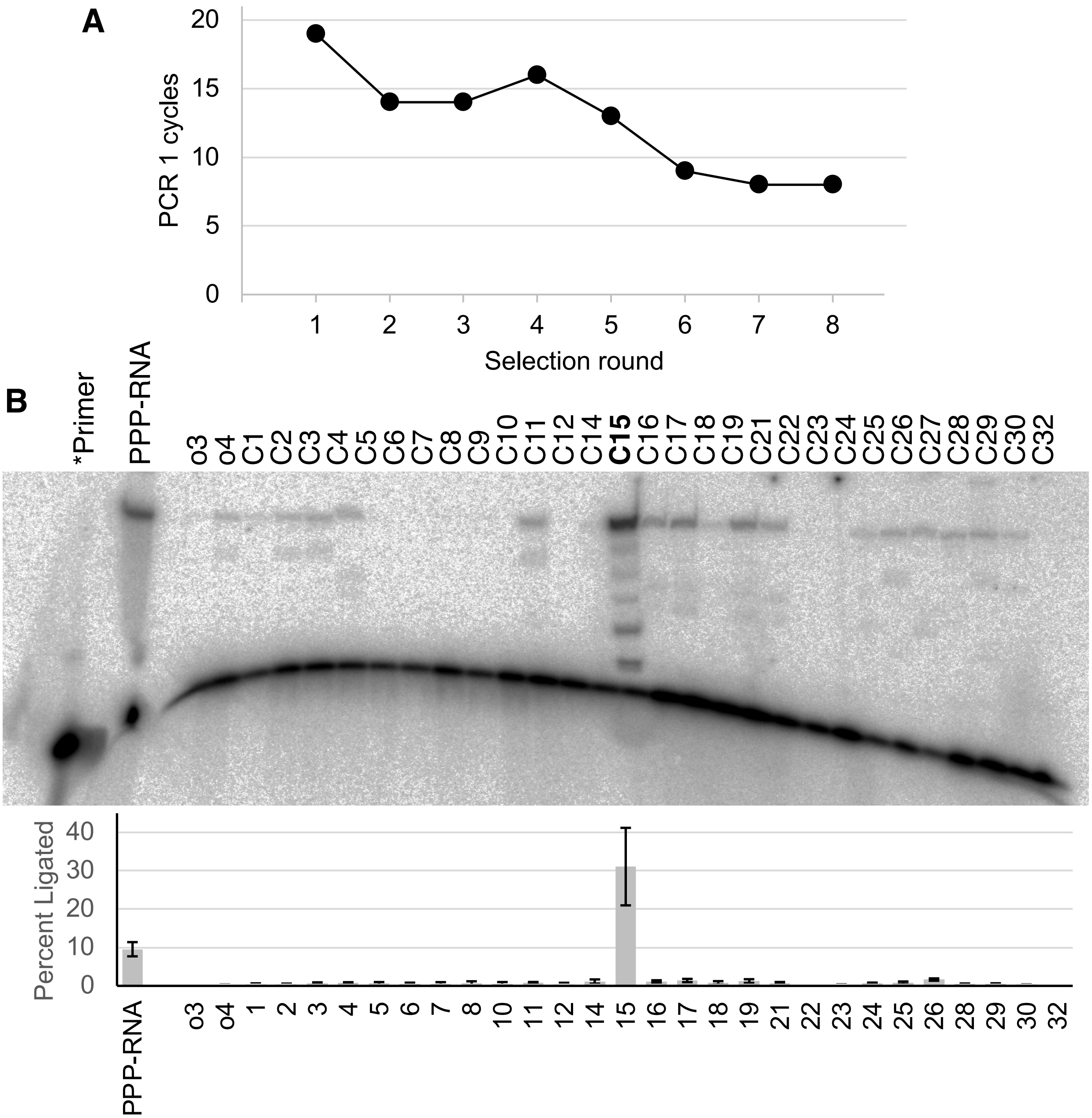

Figure S2. Detection of self-triphosphorylation activity in a library selected in the presence of Yb^{3+} from a completely randomized N_{150} sequence. **(A)** Number of PCR cycles during the selection procedure that were required to amplify selected reverse transcription products after the selection step. This cycle number was estimated from the intensity of PCR product bands on agarose gels of the correct size, with clear but not saturated intensity. **(B)** Biochemical activity of individual ribozyme clones that were arbitrarily chosen from the library after selection round 8. Clones o3 and o4 stem from an earlier selection round, while the 29 clones C1 to C32 stem from selection round 8. The ribozymes were incubated at room temperature for 3 hours in the presence of 6 mM Yb^{3+}, 6 mM cTmp, 50 mM Tris/HCl pH 7.3, and 55 mM NaCl. After ligation of 5'-triphosphorylated ribozymes to a radiolabeled primer using the R3C ligase ribozyme, products were separated by denaturing 20% PAGE and exposed to phosphorimager screens.

Figure S3

Ribozyme	Activity (% ligated)	Standard deviation	Sequence
51	16.2	4.5	GAGACCGAGATGTTCCTCACGGCCAGATTGGCTTTATACGACCACCGTTCACCTACCTTTGTTAAGTGTCTTGTGATCGCCAGTTAAGCTCCAGC
52	13.5	5.2	GAGACCGAGATGTTCCTCACGGCCAGATTGGCTTTATACGACCACCGTTCACTTCTGTTGCTAGAAGTGATTGTGATCGCCAGTTAAGCTCCAGC
42	12.6	4.7	GAGACCGAGATGTTCCTCACGGCCAGATTGGCTTTATACGACCACCGTTCACGAGTGTGTCCGACTCGCCCAGTGATCGCCAGTTAAGCTCCAGC
50	12.0	2.8	GAGACCGAGATGTTCCTCACGGCCAGATTGGCTTTATACGACCACCGTTCACGGCTGCCTATGGTTACCCCGGTGATCGCCAGTTAAGCTCCAGC
48	10.9	3.5	GAGACCGAGATGTTCCTCACGGCCAGATTGGCTTTATACGACCACCGTTCACAGGTCCACTTGCTGTATACCGTGATCGCCAGTTAAGCTCCAGC
57	10.4	5.7	GAGACCGAGATGTTCCTCACGGCCAGATTGGCTTTATACGACCACCGTTCACCTTGTACATAAGACGGATGTGTGATCGCCAGTTAAGCTCCAGC
59	9.8	6.4	GAGACCGAGATGTTCCTCACGGCCAGATTGGCTTTATACGACCACCGTTCACCGTTGCCTAATAGTGATACCGTGATCGCCAGTTAAGCTCCAGC
53	9.4	3.4	GAGACCGAGATGTTCCTCACGGCCAGATTGGCTTTATACGACCACCGTTCACAATCATTTGTGGATGCCCTTGTGATCGCCAGTTAAGCTCCAGC
55	9.3	3.8	GAGACCGAGATGTTCCTCACGGCCAGATTGGCTTTATACGACCACCGTTCACCGGATTGTTGATCTAGACGTGTGATCGCCAGTTAAGCTCCAGC
4	9.0	3.8	GAGACCGAGATGTTCCTCACGGCCAGATTGGCTTTATACGACCACCGTTCACGACTTCTCGAGGTTGTGGGGGTGATCGCCAGTTAAGCTCCAGC
60	7.4	4.4	GAGACCGAGATGTTCCTCACGGCCAGATTGGCTTTATACGACCACCGTTCACGTTGTGTTGCCACAGAACGTGTGATCGCCAGTTAAGCTCCAGC
5	7.0	2.0	GAGACCGAGATGTTCCTCACGGCCAGATTGGCTTTATACGACCACCGTTCACTTCTCCTCTTGGGTAGGTTGGTGATCGCCAGTTAAGCTCCAGC
58	7.0	4.1	GAGACCGAGATGTTCCTCACGGCCAGATTGGCTTTATACGACCACCGTTCACAGCCTTCGGGTCGTAGAAACGTGATCGCCAGTTAAGCTCCAGC
54	6.4	3.3	GAGACCGAGATGTTCCTCACGGCCAGATTGGCTTTATACGACCACCGTTCACTGCGTTGGGTTGCGTGTCGGGTGATCGCCAGTTAAGCTCCAGC
9	6.3	3.1	GAGACCGAGATGTTCCTCACGGCCAGATTGGCTTTATACGACCACCGTTCACCGTTATAGGTGCCGATGTCTGTGATCGCCAGTTAAGCTCCAGC
65	5.7	2.3	GAGACCGAGATGTTCCTCACGGCCAGATTGGCTTTATACGACCACCGTTCACAGTTTCCACGCCTGTGTTGTGTGATCGCCAGTTAAGCTCCAGC
62	4.8	2.5	GAGACCGAGATGTTCCTCACGGCCAGATTGGCTTTATACGACCACCGTTCACGGCTTTGATGGTTGCGGAAGGTGATCGCCAGTTAAGCTCCAGC
70	3.5	1.6	GAGACCGAGATGTTCCTCACGGCCAGATTGGCTTTATACGACCACCGTTCACATGTTTATGCACCATTCCGGGTGATCGCCAGTTAAGCTCCAGC
67	3.1	1.6	GAGACCGAGATGTTCCTCACGGCCAGATTGGCTTTATACGACCACCGTTCACCTGCAGGGATATGCGTGAGGGTGATCGCCAGTTAAGCTCCAGC
73	2.2	1.6	GAGACCGAGATGTTCCTCACGGCCAGATTGGCTTTATACGACCACCGTTCACTCAGTTTTCGTGTGTACGTCGTGATCGCCAGTTAAGCTCCAGC
61	0.7	0.2	GAGACCGAGATGTTCCTCACGGCCAGATTGGCTTTATACGACCACCGTTCACGTCTCCAGCTCCCTGCTTTCGTGATCGCCAGTTAAGCTCCAGC
66	0.5	0.1	GAGACCGAGATGTTCCTCACGGCCAGATTGGCTTTATACGACCACCGTTCACCCAAATAGACGAATTGGTTTGTGATCGCCAGTTAAGCTCCAGC
8	0.2	0.1	GAGACCGAGGATGTTCCTCACGGCCAGATTGGCTTTATACGACCACCGTTCACTTCGGGCGTGAAGTGTATTCGTGATCGCCAGTTAAGCTCCAGC

Figure S3. Activity and sequences of 23 clones with an N20 sequence replacing nucleotides 54-158. Reactions were performed at 6 mM Yb^{3+}, 6 mM cTmp, 50 mM HEPES/KOH pH 7.3, 150 mM NaCl, for 3 hours at 22°C. Each row describes one ribozyme clone, with the clone names in the left column. The rows are sorted based on their activity (second column), which lists the 'percent ligated' with the R3C ligase ribozyme, as a measure of its self-triphosphorylation activity. Values are averages of triplicate experiments, with the third column showing their standard deviation. The complete ribozyme sequences are listed in column four, with the portion of the randomized N20 sequence highlighted. Ribozyme 8 contains a nucleotide insertion (blue highlight), which may contribute to its low activity.

Figure S4

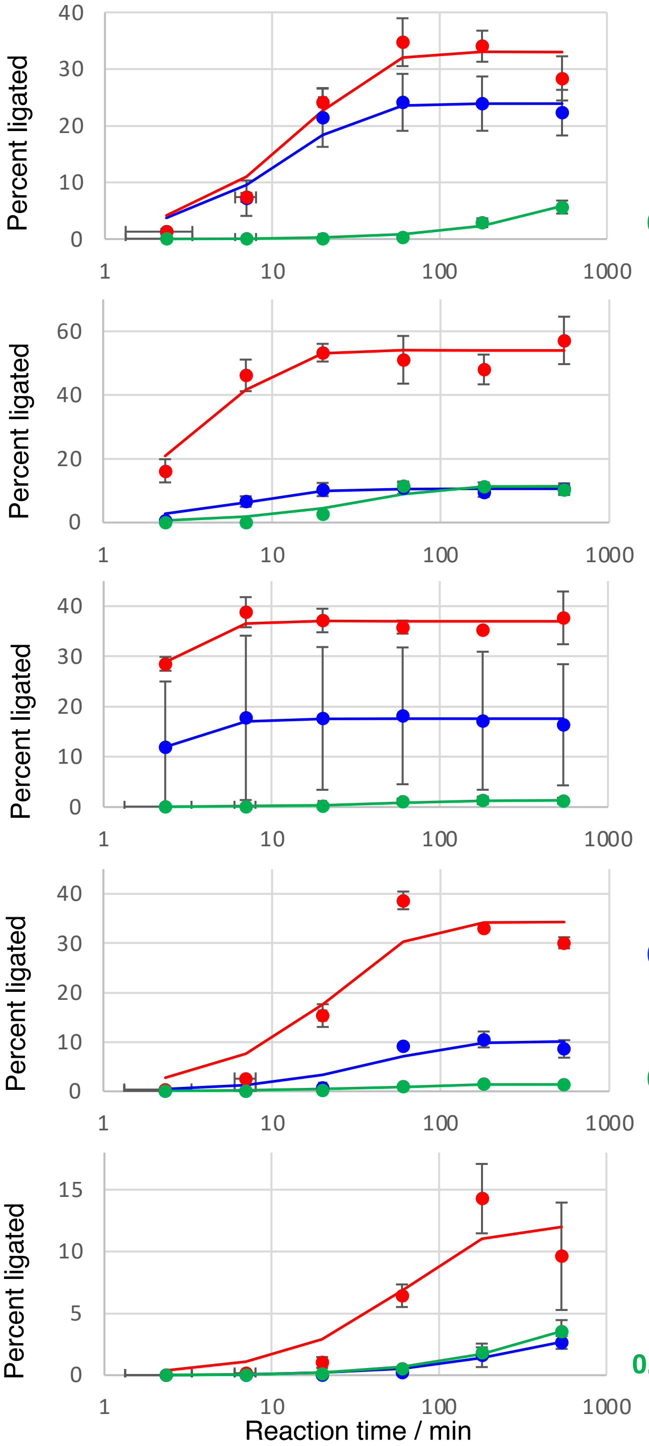

[cTmp]	[Yb^{3+}]	Rate	Amplitude
1 mM	0.33 mM	0.073 min^{-1}	24 %
1 mM	0.1 mM	0.058 min^{-1}	33 %
0.33 mM	1 mM	0.0010 min^{-1}	14 %
3.3 mM	1 mM	0.13 min^{-1}	11 %
3.3 mM	0.33 mM	0.21 min^{-1}	54 %
1 mM	1 mM	0.025 min^{-1}	11 %
10 mM	3.3 mM	0.49 min^{-1}	18 %
10 mM	1 mM	0.65 min^{-1}	37 %
3.3 mM	3.3 mM	0.018 min^{-1}	1.4 %
0.33 mM	0.33 mM	0.020 min^{-1}	10 %
0.3 mM	0.1 mM	0.036 min^{-1}	34 %
0.33 mM	0.033 mM	0.018 min^{-1}	1.4 %
0.1 mM	0.33 mM	0.0029 min^{-1}	3.4 %
0.1 mM	0.1 mM	0.014 min^{-1}	12 %
0.033 mM	0.1 mM	0.0024 min^{-1}	4.9 %

Figure S4. Kinetic data obtained by single exponential curve fitting to reactions with varying concentrations of Mg^{2+} and cTmp. Only those combinations of cTmp concentration and Yb^{3+} concentration that led to at least 1% of the ribozyme being ligated were used in this analysis to avoid problems stemming from a low signal-to-noise ratio. Data are averages from three experiments (filled circles), with error bars indicating standard deviations. The large error bars of the data set 10 mM cTmp / 3.3 mM Yb^{3+} are not problematic for rate determination because each of the three individual experiments had a similar rate – only the amplitudes differed between the replicates. The concentrations of cTmp and Yb^{3+} in all reactions is labeled next to each graph.

Figure S5

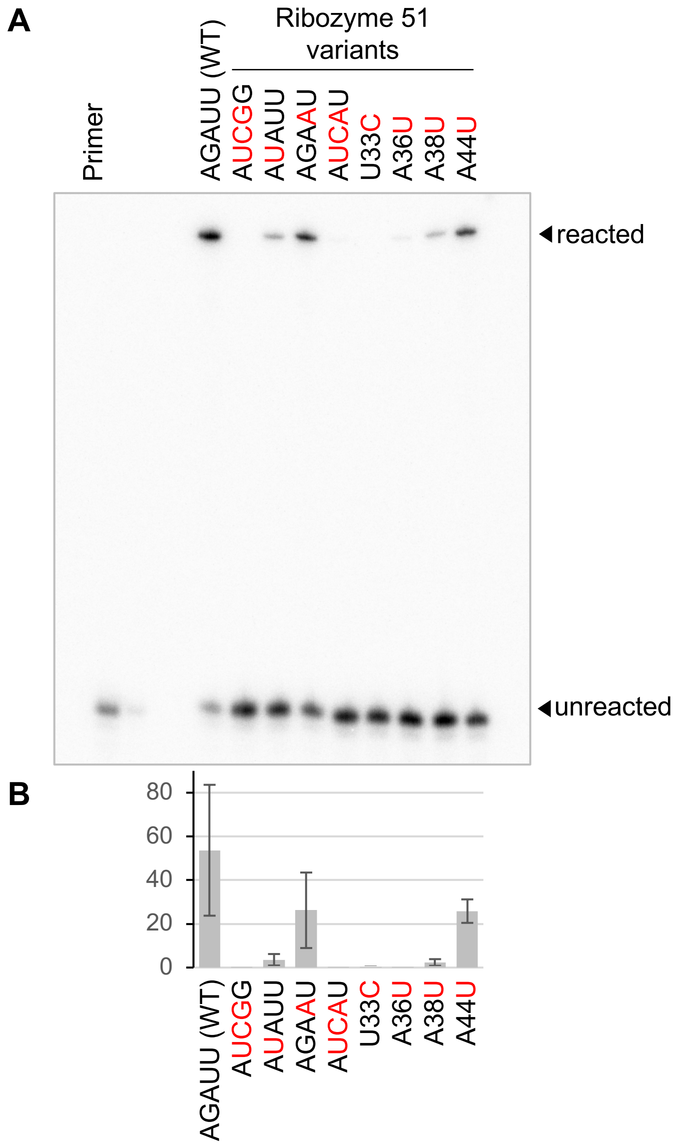

Figure S5: Supplementary details for Figure 7 in the main manuscript, on the activity of mutation variants of ribozyme 51 positions 25-29, and three bulged adenosines at positions 36, 38, and 44. **(A)** Phosphorimage of products from the ligation assay of ribozyme 51 variants with a 5'-[^{32}P] radiolabeled 16mer RNA, after separation by denaturing 10% polyacrylamide gel electrophoresis. The position of the unreacted 16mer and the reacted, gel-shifted 111-mer are indicated. The fraction of shifted RNA reports on the fraction of shifted ribozyme because these two reaction partners were employed at equimolar concentrations. **(B)** Graph of the fraction of gel-shifted RNA 16-mer for the Wild-Type ribozyme 51 (loop sequence 25-29 of AGAUU), four variants with mutations in that loop sequence, and four variants with point mutations in the ribozyme. The mutated positions are highlighted in red. The mutation U33C converted a GU pair to a GC pair at the basis of the stem-loop containing the loop 25-29. While the effect of this mutation was surprisingly strong it was not described in the main manuscript because it did not fit the rationale of the storyline. Error bars are the standard deviations from three experiments. The large error bars of the WT and mutant AGAAU are not a concern because one replicate gave a high value for both variants, and another replicate gave a low value for both variants, therefore the ratio between both variants was similar.

Figure S6

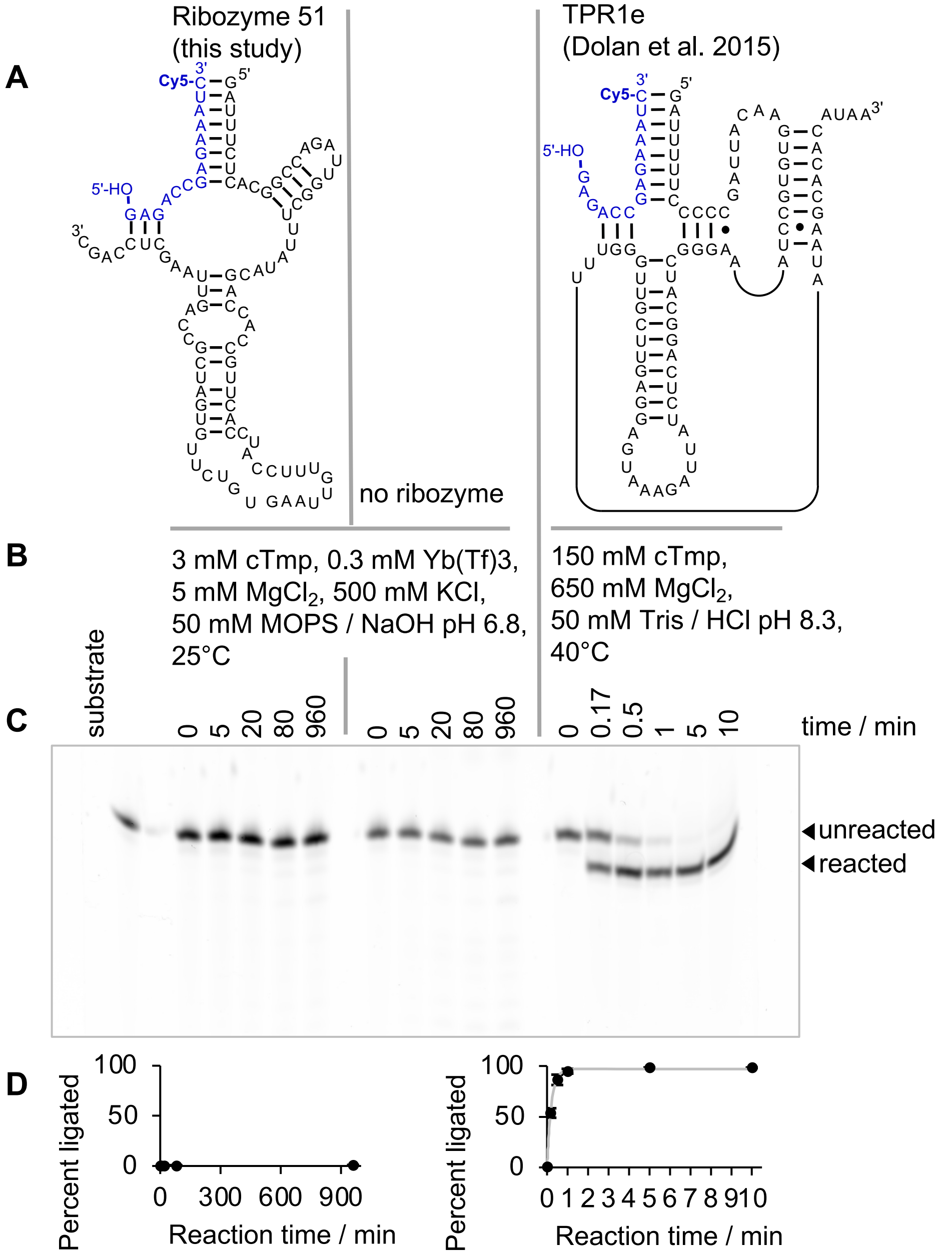

Figure S6: Test of the lanthanide-using ribozyme 51 for its potential to trans-triphosphorylate a short RNA. **(A)** The secondary structure of ribozyme 51 is shown, alongside the secondary structure of the ribozyme TPR1e, which was used as positive control. The short substrates used in trans are shown in blue, with a 3'-Cy5 label that was added using pCp-Cy5 (Jena Biosciences) and T4 RNA ligase. **(B)** Optimized reaction conditions for the lanthanide using ribozyme (left) and the Mg^{2+} using ribozyme (right). **(C)** Scan of reaction products with excitation at 635 nm (Typhoon imager) that were separated by 7M urea 20% PAGE. Note that the weak, fuzzy band appearing with the lanthanide ribozyme was not due to triphosphorylation but due to degradation, as shown by an incubation of the substrate in the optimized reaction buffer without the lanthanide-using ribozyme. **(D)** Graphs showing the observed reaction kinetics of ribozyme 51, where no detectable signal was found after 16 hours of incubation, and TPR1e. The data from TPR1e were best fitted with a single-exponential function to a maximum of 97% and k_{OBS} = 4.7 min^{-1}, which is close to the 6.8 min^{-1} observed for the corresponding cis-reacting TPR1e under the same reaction conditions (Dolan et al 2015). Symbols indicate averages from three experiments, and error bars are their standard deviations. If error bars are not visible they are smaller than the symbols.
